# An Artificial Synaptic Device Based on InSe/Charge Trapping Layer/h-BN Heterojunction with Controllable Charge Trapping via Oxygen Plasma Treatment

**DOI:** 10.3390/mi16121422

**Published:** 2025-12-18

**Authors:** Qinghui Wang, Jiayong Wang, Manjun Lu, Tieying Ma, Jia Li

**Affiliations:** 1 School of Electrical and Information Technology, Yunnan Minzu University, Kunming 650500, China; 2 Yunnan Key Laboratory of Unmanned Autonomous System, Kunming 650500, China; 3 Institute of Integrated Circuits, Shanghai University, Shanghai 201800, China; wangjiayong@shu.edu.cn; 4 Shaanxi Institute of Metrology Sciences, Xi’an 710000, China; lucifer998@163.com; 5 College of Opitical and Electronic Technology, China Jiliang University, Hangzhou 310018, China; mty@cjlu.edu.cn

**Keywords:** neuromorphic computing, InSe, artificial synaptic devices, charge trapping layer, heterojunction

## Abstract

Neuromorphic computing, an emerging computational paradigm, aims to overcome the bottlenecks of the traditional von Neumann architecture. Two-dimensional materials serve as ideal platforms for constructing artificial synaptic devices, yet existing devices based on these materials face challenges such as insufficient stability. Indium selenide (InSe), a two-dimensional semiconductor with unique properties, demonstrates significant potential in the field of neuromorphic devices, though its application research remains in the initial stage. This study presents an artificial synaptic device based on the InSe/Charge Trapping Layer (CTL)/h-BN heterojunction. By applying oxygen plasma treatment to h-BN to form a controllable charge-trapping layer, efficient regulation of carriers in the InSe channel is achieved. The device successfully emulates fundamental synaptic behaviors including paired-pulse facilitation and long-term potentiation/inhibition, exhibiting excellent reproducibility and stability. Through investigating the influence of electrical pulse parameters on synaptic weights, a structure–activity relationship between device performance and structural parameters is established. Experimental results show that the device features outstanding linearity and symmetry, realizing the simulation of key synaptic behaviors such as dynamic conversion between short-term and long-term plasticity. It possesses a high dynamic range ratio of 7.12 and robust multi-level conductance tuning capability, with stability verified through 64 pulse cycle tests. This research provides experimental evidence for understanding interfacial charge storage mechanisms, paves the way for developing high-performance neuromorphic computing devices, and holds broad application prospects in brain-inspired computing and artificial intelligence hardware.

## 1. Introduction

Neuromorphic computing, an emerging paradigm to break through the bottleneck of the von Neumann architecture, realizes in-memory computing by mimicking biological synaptic plasticity, providing a new pathway for low-power and efficient information processing. Two-dimensional (2D) materials, with their atomic-layer thickness, tunable electronic structure, and van der Waals integration properties, have become ideal platforms for constructing artificial synaptic devices [1,2].

Significant progress has been made in synaptic devices based on 2D heterojunctions. For example, heterojunction devices such as ReS_2_/h-BN/graphene [3] and WSe_2_/MoTe_2_ [4] have successfully simulated core synaptic behaviors including long-term potentiation/depression (LTP/LTD) and spike-timing-dependent plasticity (STDP)—a typical form of long-term plasticity based on LTP/LTD. However, synaptic devices based on two-dimensional materials, including transition metal dichalcogenides (TMDCs), generally face challenges such as insufficient stability and limited regulation accuracy. For instance, black phosphorus (BP), a single-element two-dimensional material, suffers from short device lifetimes due to easy oxidation, and MXene (a type of two-dimensional transition metal carbide/nitride) is constrained by complex preparation processes and interfacial defects, making high-precision weight modulation difficult to achieve.

InSe, a 2D semiconductor with a high carrier mobility exceeding 10^4^ cm^2^/(V·s), enables precise control of carrier transport more easily through van der Waals heterojunction integration [5,6]. Nevertheless, the application of InSe in neuromorphic devices is still in its infancy, and how to leverage its unique high carrier mobility to optimize synaptic behavior regulation mechanisms remains a critical scientific problem to be explored.

To address these challenges, this study designed an InSe/CTL/h-BN van der Waals heterojunction synaptic device. By treating the h-BN layer with oxygen plasma, controllable atomic-scale defect sites were constructed at the interface as charge trapping centers, achieving efficient modulation of the carrier concentration in the InSe channel. The device could stably simulate basic synaptic behaviors such as paired-pulse facilitation [7] and long-term potentiation/depression [8]. Through systematic investigation of the quantitative relationships between electrical pulse parameters (amplitude, width, frequency) and synaptic weights, a structure–property relationship model linking device parameters (trapping layer thickness, InSe layer number) and performance metrics is established, providing theoretical guidance for the large-scale fabrication of InSe-based devices. Compared with reported 2D synaptic devices, this work achieves important breakthroughs in stability and functional integration, offering a new material system and device development concept for constructing high-performance brain-inspired computing systems.

## 2. Materials and Methods

**Figure 1:**
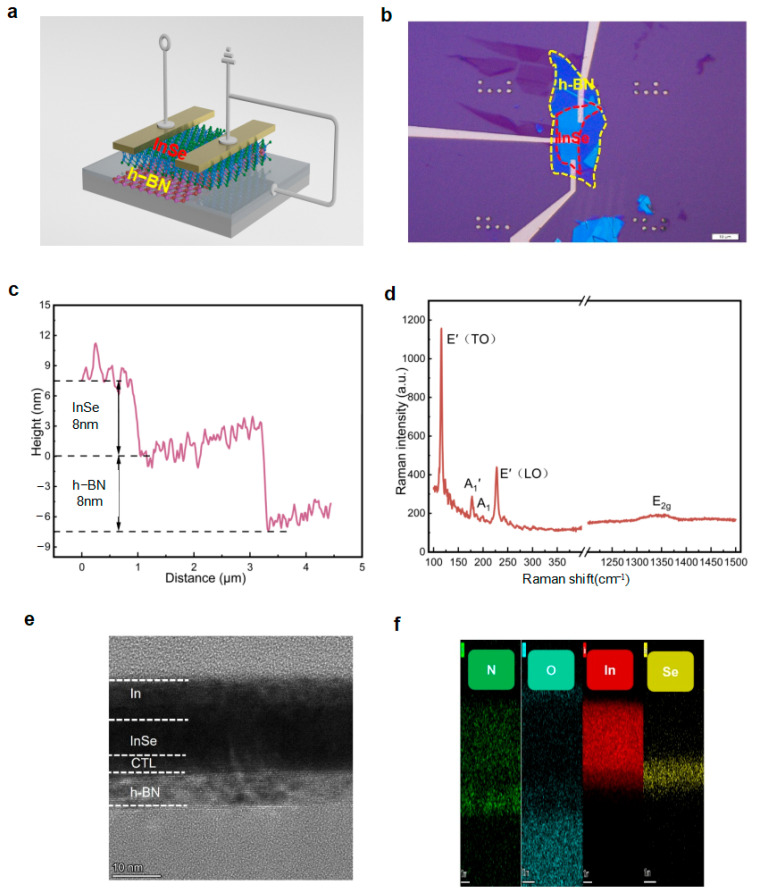
(**a**) Schematic diagram of the InSe/CTL/h-BN heterojunction device; (**b**) optical microscopy image of the device structure; (**c**) AFM topography characterization of the device thickness; (**d**) Raman spectroscopy characterization; (**e**) Transmission Electron Microscopy (TEM); (**f**) EDS analysis.

We successfully fabricated a charge-trapping neuromorphic device based on InSe, which employs h-BN as the substrate, oxygen-plasma-treated h-BN as the CTL, and InSe as the channel layer. As shown in Figure 1a, the device fabrication process involves four key steps: first, transferring h-BN onto a cleaned Si/SiO_2_ substrate via the mechanical exfoliation transfer method; subsequently, performing oxygen plasma treatment on the h-BN surface to form the CTL layer using reactive ion etching at a power of 150 W, oxygen flow rate of 35 sccm, processing time of 300 s, and pressure of 110 mTorr; then, transferring the InSe thin layer to construct the heterojunction; and finally, completing the preparation of In electrodes through electron-beam evaporation, photolithography, and magnetron sputtering processes [9]. The 100× optical microscopy image in Figure 1b shows clear interfaces and good contact between layers without bubbles or impurities.

Systematic analysis of the device was conducted using multiple characterization techniques. Atomic force microscopy (AFM) measurements (Figure 1c) reveal that both h-BN and InSe have a thickness of approximately 8 nm, with smooth and unbroken surfaces. Notably, the 10-nm-thick In electrodes exist as isolated island structures due to the failure to form a continuous film, leading to non-conductivity in the lateral direction [10]. Raman spectroscopy analysis (Figure 1d) detects characteristic peaks of InSe at 115 cm^−1^, 177 cm^−1^, 200 cm^−1^, and 227 cm^−1^ (A_1_′, E′ (TO), E′ (LO), and A_1_ modes), along with the h-BN characteristic peak at 1367 cm^−1^, with no significant shifts in peak positions, confirming good heterojunction interface quality and no obvious lattice distortion. Further high-resolution transmission electron microscopy (HRTEM) and energy-dispersive X-ray spectroscopy (EDS) analysis (Figure 1e) show significant oxygen element distribution in both the In surface layer and the h-BN surface layer: oxidation in the In layer originates from air exposure, while the oxygen distribution in the h-BN layer confirms successful introduction of charge-trapping sites via plasma treatment. The layer thicknesses measured by TEM are consistent with the AFM results.

## 3. Results and Discussion

**Figure 2:**
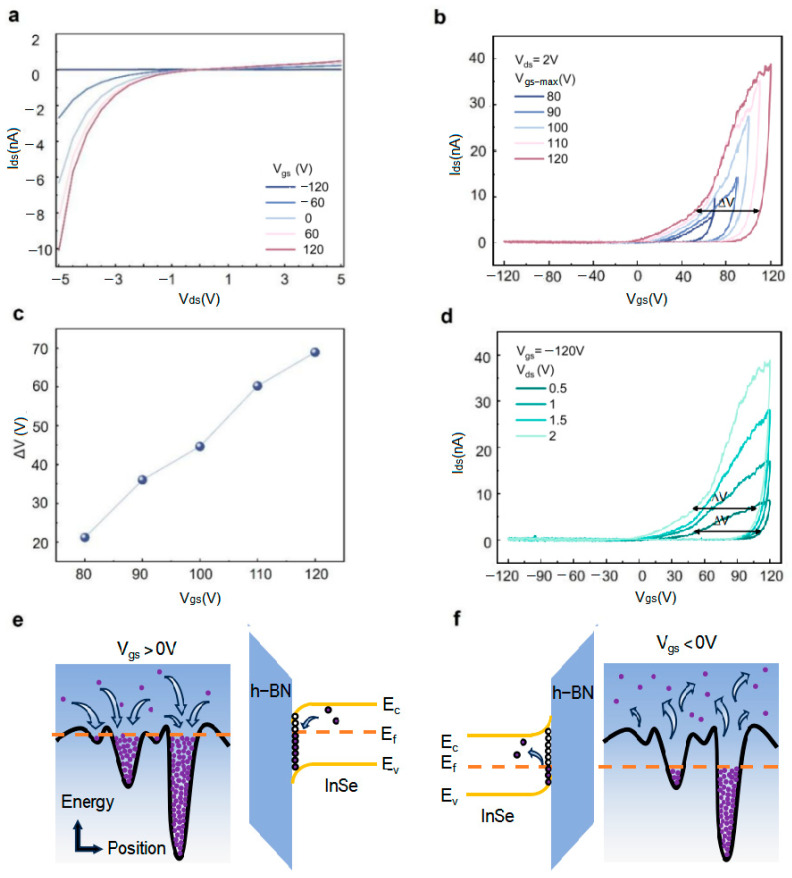
Electrical transport characteristics and principal analysis of the InSe/CTL/h-BN heterojunction transistor device: (**a**) Output characteristics curves; (**b**) Transfer characteristics curves; (**c**) Memory window variation with back-gate voltage; (**d**) Influence of source-drain voltage on the memory window; (**e**) Energy band diagram of the electron trapping and release principle under positive gate voltage; (**f**) Energy band diagram of the electron trapping and release principle under negative gate voltage.

Electrical transport characteristics were characterized through systematic electrical measurements. Figure 2a displays the output characteristics curves (I_ds_–V_ds_) ranging from −120 V to 120 V, with a step amplitude of 60 V. Experimental data showed that the device successfully conducts under the test conditions, generating a channel current (I_ds_) on the nanometer scale. Notably, the output characteristics exhibit typical van der Waals contact features, and the channel current demonstrates a significant gate-voltage modulation effect.

To investigate the influence of back-gate voltage on the device’s conductivity characteristics, bidirectional gate-voltage sweep tests were performed at a fixed source-drain voltage (V_ds_ = 2 V), as shown in Figure 2b. The test adopted a scanning mode from negative to positive gate voltage (forward sweep) and then back to negative gate voltage (reverse sweep). The obtained transfer characteristics curves (I_ds_–V_gs_) clearly reflect the dynamic change process of carrier concentration in the channel layer. The curve exhibits typical clockwise hysteresis characteristics and n-type semiconductor behavior. Quantitative analysis indicates that as the gate-voltage scanning range increases, the device’s conductivity systematically enhances, and the memory window of the hysteresis curve expands accordingly, reaching a maximum of approximately 130 V—corresponding to stronger charge storage capacity and superior non-volatile memory characteristics.

Further research (Figure 2c) revealed a positive correlation between the memory window of the InSe/CTL/h-BN heterostructure device and the amplitude of the applied back-gate voltage, a regular variation that clearly confirms the presence of significant charge trapping behavior in the device. By systematically measuring transfer characteristics under different gate-voltage scanning ranges, we quantitatively characterized the evolution of the memory window. Experimental results showed that the device exhibits a prominent memory window within specific gate-voltage operating intervals, a feature that highlights its important potential for non-volatile memory and artificial synaptic applications. Additionally, the data in Figure 2d confirm that the memory window ΔV is insensitive to changes in source-drain voltage—a key finding indicating that the number of charges in the channel layer is primarily regulated by the gate electric field rather than the source-drain electric field.

The charge trapping and release behavior of the device can be deeply interpreted through band engineering. As shown in the left panel of Figure 2e, under a positive gate voltage, electrons in the channel layer are captured by defect states. The applied positive gate voltage elevates the Fermi level E_F_ of the InSe channel layer, bringing it closer to the conduction band (right panel of Figure 2e). Due to the special treatment of the h-BN interface, the CTL contains abundant oxygen vacancy defect states, which capture electrons from the InSe channel under the positive gate voltage. This process reduces the concentration of free carriers in the channel, thereby decreasing conductivity. Conversely, when a negative gate voltage is applied (Figure 2f), the Fermi level of InSe decreases, moving closer to the valence band. Electrons trapped by oxygen vacancy defect states at the CTL interface gain sufficient energy under the strong electric field and are released back into the InSe channel layer, increasing channel conductivity. This charge trapping–release process exhibits distinct hysteresis characteristics, causing the device’s conductance state to change nonlinearly with gate-voltage scanning.

This gate-controlled charge storage mechanism closely mimics the weight regulation behavior of biological synapses: a positive gate voltage (analogous to synaptic depression stimuli) induces charge trapping, reducing conductance to simulate LTD; a negative gate voltage (analogous to synaptic potentiation stimuli) promotes charge release, increasing conductance to simulate LTP. Therefore, the device effectively emulates biological synaptic plasticity, providing a reliable hardware implementation for neuromorphic computing.

**Figure 3:**
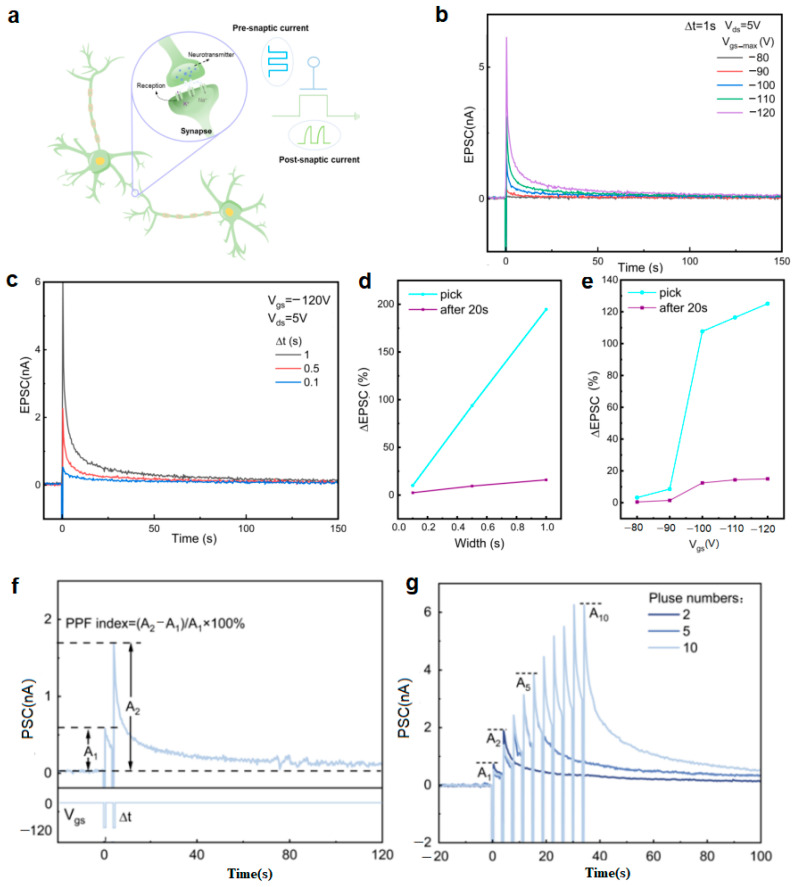
Electrical synaptic plasticity tests based on InSe: (**a**) Schematic diagrams of a biological synapse (**left**) and an artificial synapse (**right**); (**b**) EPSC responses under single-pulse stimulation with pulse amplitudes from −80 V to −120 V and a pulse interval of 1 s; (**c**) EPSC responses under electrical pulse stimulation with a fixed amplitude of −120 V and pulse intervals from 0.1 s to 1 s; (**d**) Relationship between synaptic weight and pulse interval; (**e**) Relationship between synaptic weight and pulse amplitude. Synaptic behavior tests of the InSe-Based artificial synaptic device: (**f**) PPF triggered by two consecutive pulses with an amplitude of −120 V, width of 100 ms, and interval of 250 ms; (**g**) Relationship between the PSC and the number of multi-pulse electrical stimulations (2, 5, and 10 pulses) with a fixed amplitude of −120 V, width of 100 ms, and interval of 250 ms.

The dynamic response characteristics of the artificial synaptic device (Figure 3a) were systematically investigated through electrical testing [11]. As shown in Figure 3b, under a fixed pulse width (Δt = 1 s), both the peak and the steady-state values of the excitatory postsynaptic current (EPSC) increased significantly as the pulse amplitude increased from −80 V to −120 V. Notably, when the pulse amplitude reached −120 V, the current decay time was significantly prolonged, indicating a gradual transition from short-term potentiation (STP) [12,13] with rapid recovery to LTP [8,14] with persistent stability. This transition behavior arises from high-energy pulses inducing more electrons to be trapped at the CTL interface, forming more stable charge storage states. Similar transition patterns were observed in pulse width modulation experiments (Figure 3c). At a fixed pulse amplitude (−120 V), increasing the pulse width from 0.1 s to 1 s not only enhanced the EPSC amplitude but also significantly slowed its decay dynamics. Quantitative analysis (Figure 3d,e) revealed that both increasing pulse amplitude and prolonging pulse width led to elevated synaptic weight (ΔW = ΔI/I) and prolonged decay time. This tunable response characteristic closely mimics the intensity-dependent plasticity of biological synapses, where strong stimuli induce more persistent changes in synaptic efficacy.

The experimental observations can be explained by charge trapping dynamics: high-energy pulses (large amplitude or long duration) facilitate more extensive charge exchange processes at the CTL interface. During pulse application, a large number of electrons are trapped in deep energy level sites; after pulse removal, these trapped charges require longer times to be released via thermal excitation or tunneling mechanisms, manifesting as slow current decay and LTP behavior.

The short-term plasticity characteristics of the artificial synaptic device were systematically investigated through paired-pulse experiments [7]. As shown in Figure 3f, when a pair of negative gate pulses (−120 V, 100 ms interval) was applied, the post-synaptic current (PSC) exhibited a significant enhancement effect because the current induced by the first pulse had not fully decayed when the second pulse was applied [15]. This paired-pulse facilitation (PPF) phenomenon demonstrated a typical pulse interval dependence, with the PPF index increasing significantly as Δt decreased [16]. Further investigation revealed that the synaptic plasticity state could be effectively regulated by increasing the number of consecutive pulses. Figure 3g shows that as the number of pulse stimuli accumulated, the PSC response gradually transitioned from short-term potentiation with rapid decay to LTP with persistent stability. This transition process was closely related to the pulse-induced charge trapping dynamics: consecutive pulse stimuli promoted progressive charge accumulation at the CTL interface, ultimately forming a stable charge storage state. The experimental results indicate that the device can not only simulate the short-term plasticity behavior of biological synapses but also achieve a controllable transition to long-term plasticity through pulse sequence regulation. These characteristics are attributed to the device’s unique charge trapping mechanism: on a short time scale, consecutive pulses lead to the gradual filling of interface trap energy levels; on a long time scale, trapped charges are slowly released through the thermal excitation process.

**Figure 4:**
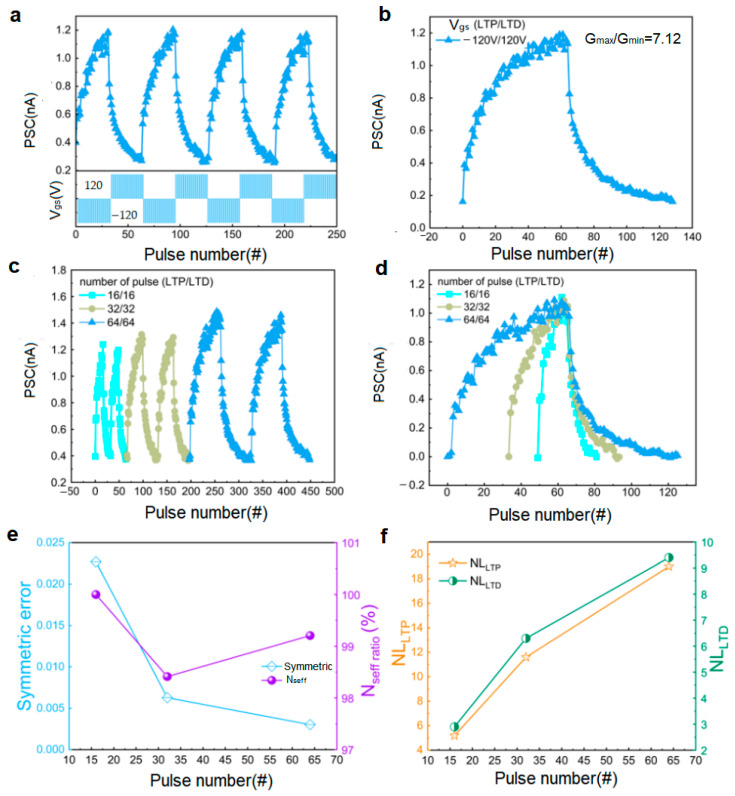
Synaptic behavior tests of the InSe-Based artificial synaptic device: (**a**) LTP/LTD curves of the artificial synaptic device, where 64 consecutive −120 V Vg pulses followed by 64 + 120 V V_g_ pulses constitute one cycle, applied for 4 consecutive cycles; (**b**) Extracted LTP/LTD curve of a stable cycle; (**c**) LTP/LTD curves of the device under different pulse numbers, with 2 cycles per pulse count; (**d**) Normalized LTP/LTD curves for different pulse numbers; (**e**) Extracted symmetric error and *N*_seff_; (**f**) Extracted NL.

The long-term plasticity characteristics of the device were explored through systematic experiments. As shown in Figure 4a, applying four cycles of continuous pulse stimulation (each cycle containing 64 negative pulses at −120 V and 64 positive pulses at 120 V) revealed stable LTP/LTD cyclic characteristics. Under negative pulse stimulation, the device conductance continuously increased (LTP); subsequently, under positive pulse stimulation, the conductance gradually decreased and recovered to the initial value (LTD). Data from the stable operating interval (Figure 4b) show that the dynamic range of the PSC is 1.63 × 10^−1^–1.16 nA with a dynamic range ratio of 7.12, demonstrating excellent multi-level conductance states. The influence of the number of pulses on plasticity was further investigated. Figure 4c compares LTP/LTD characteristic curves with 16, 32, and 64 pulses per half-cycle (all with a pulse amplitude of 120 V). Experimental results show that the PSC response amplitude significantly increases with the number of pulses. To analyze this pattern more clearly, we normalized the single-cycle data (Figure 4d). Based on these results, we used the nonlinearity (NL) to quantitatively evaluate the device’s plasticity characteristics, with the following calculation formula [17,18]:

(1) $$G_{LTP}=G_{c}(1-e^{-\frac{P}{L}})+G_{\min}$$

(2) $$G_{LTD}=-G_{c}(1-e^{\left(\frac{P-P_{\max}}{L}\right)})+G_{\max}$$

(3) $$G_{c}=(G_{\max}-G_{\min})/(1-e^{\left(-\frac{P_{\max}}{L}\right)})$$

Here, *G_LTP_* and *G_LTD_* represent the conductance of the long-term potentiation and long-term depression curves, respectively; *G_c_* is the fitting constant for normalized conductance; *L* is the numerical value of NL; *P* is the number of applied pulses; *P*_max_ is the maximum number of pulses; and *G*_max_ and *G*_min_ are the maximum and minimum conductance values, respectively. Taking the LTD process as an example, experimental results (Figure 4e) show: (1) In the initial stage (with fewer pulses), the number of charges trapped in the trap energy levels is limited, leading to a small decrease in PSC; (2) as the number of pulses increases, more charges are trapped, causing a significant decrease in conductivity and a larger reduction in PSC; (3) in the later stage (at high pulse numbers), due to most traps being occupied by charges, a local charge shielding effect occurs, slowing the rate of PSC decrease. NL analysis indicates that the NL value gradually increases with the number of pulses (Figure 4f), indicating a decrease in device stability. This phenomenon is closely related to the state saturation effect in the charge trapping process: a continuous increase in pulse stimulation leads to the gradual filling of trap energy levels, causing the device response to tend toward nonlinearity and reflecting the inherent physical limitations of the charge storage mechanism. The symmetry is the reciprocal of the symmetry error, defined by the following formula [19]:

(4) $$symmetric\;error=\sum_{k=1}^{n}\frac{{\left(G_{N}(k)-G_{N}(2n-k)\right)}^{2}}{n}$$

(5) $$N_{\mathrm{seff}}=\mathrm{count}\{\frac{\Delta G}{G_{\max}-G_{\min}}>0.005\}$$

Here, *G_N_* (*k*) represents the normalized conductance, *n* is the number of pulses per half-cycle, and *N*_seff_ is the number of discrete conductance states when the ratio of Δ*G* to the (*G*_max_ − *G*_min_) exceeds 0.005 [17,19]. As shown in Figure 4e, the relationship between symmetry, *N*_seff_, and the number of pulses indicates that the device exhibits higher stability with fewer pulses.

## 4. Conclusions

In this study, we successfully constructed a charge-trapping artificial synaptic device based on InSe, achieving precise simulation of biological synaptic behaviors. Through oxygen plasma treatment, a controllable CTL was engineered at the interface between h-BN and InSe. This innovative design enables the device to effectively regulate conductance changes in the channel layer via the CTL and achieve precise tuning of the memory window. Experimental results demonstrate that the device not only exhibits excellent linearity and symmetry but also successfully emulates key synaptic behaviors, including dynamic conversion between STP and LTP, PPF effect, and LTP/LTD. Systematic investigations into the influence of pulse parameters reveal that the device possesses a dynamic range ratio as high as 7.12 and robust multi-level conductance modulation capability, with 64-pulse cycle tests further confirming its outstanding stability. These findings not only provide new experimental insights into the interfacial charge storage mechanism but also open up new technical pathways for developing high-performance neuromorphic computing devices, showcasing significant application prospects in the fields of brain-inspired computing and artificial intelligence hardware.

## Data Availability

Dataset available on request from the authors.
